# The promise, perceptions, and pitfalls of immunoassays for autoantibody testing in myositis

**DOI:** 10.1186/s13075-020-02210-2

**Published:** 2020-05-15

**Authors:** Sarah L. Tansley, Julia Snowball, John D. Pauling, Anya Lissina, Masataka Kuwana, Lisa G. Rider, Johan Rönnelid, Neil J. McHugh, on behalf of the International Myositis Assessment and Clinical Studies (IMACS) Group Myositis Autoantibody Scientific Interest Group

**Affiliations:** 1 University of Bath, Bath, UK; 2 Keio University, Tokyo, Japan; 3 National Institute of Health, Bethesda, MD USA; 4 Uppsala Universitet, Uppsala, Sweden

**Keywords:** Myositis, Autoantibody

## Abstract

**Background:**

A myositis-specific autoantibody can now be identified in the majority of patients with myositis. They identify homogeneous patient subgroups and are key tools in developing a personalized approach to disease management. There is substantial clinical interest in exploiting myositis autoantibodies as biomarkers, and consequently, a large number of commercial assays have been developed for their detection. These assays are already in widespread clinical use. In order to better understand perceived concerns from the international myositis community in relation to the reliability of these assays and how they are being used, we conducted a survey of international myositis experts, all of whom were members of the International Myositis Assessment and Clinical Studies group.

**Results:**

We collected data on the types of assay used, manufacturers, and the nature of the report provided by different laboratories and received 111 complete responses. Respondents also provided information on how they used the different assays, their confidence in the results, and how this influenced their clinical practice. Enzyme immunoassay/ELISA was the most popular assay method used worldwide followed by line blot. Line blot was the most popular method used in Europe. Despite concerns from over 80% of respondents regarding false-positive and false-negative results with the assay used by their laboratory, over 80% reported that the identification of a myositis autoantibody influenced their diagnostic confidence, the information they provided to a patient, and their recommended treatment.

**Conclusions:**

In spite of ongoing concerns from the majority of users regarding the reliability of the results, myositis-specific autoantibody testing, using commercial immunoassays, is being used globally to inform clinical decision-making. These findings highlight the need for urgent guidance on the use of myositis autoantibody testing and on the interpretation of results. Knowledge of the reliability of currently available assays is essential given the importance already placed on myositis-specific autoantibodies as clinical decision-making tools.

Nearly half a century has elapsed since the identification of the first myositis-specific autoantibody (MSA) [1]. A further sixteen MSAs have subsequently been described, and MSAs are now detectable in over 60% of people with myositis [2–4]. MSAs are generally mutually exclusive and identify individual homogeneous patient subgroups. They are important prognostic biomarkers and, as such, may have a role in the development of more personalized approaches to disease management. The real “boom” period for MSA discovery was between 1999 and 2009, when over 40% of the MSAs described to date were reported [5]. Initially, these newer MSAs remained largely in the research domain as the laboratory techniques required to identify them were highly specialized, low-throughput, and expensive. The recent emergence of commercially available immunoassays to detect MSAs has increased access to these investigations, offering rapid MSA characterization, at low cost and without the need for specialist expertise. These assays were promptly adopted by clinicians and are now in widespread clinical use via commercial testing. While essential if MSAs are to be utilized in routine clinical practice, the benefits of broader access to these tools have been somewhat offset by concerns regarding the reliability of these immunoassays, specifically a low sensitivity for some key autoantibodies and a high false-positive rate in healthy controls, as highlighted by ourselves and others [6–10]. Validating new testing methods in any rare disease is a challenge, and this is further compounded by the rarity of some of the MSAs themselves. For example, certain anti-synthetase MSAs included in commercial immunoassays are found in just 0.3% of myositis patients [2].

The International Myositis Assessment and Clinical Studies (IMACS) group is a coalition of health care providers and researchers with an interest in myositis who seek to facilitate collaborative international myositis research. Recognizing both the potential benefits and pitfalls of MSA testing, IMACS established a Myositis Autoantibodies Scientific Interest Group. Given the absence of any guidance in this area, the group first sought to determine how MSA testing is being used in current clinical practice. A health professional survey was conducted to determine participants’ experience with myositis autoantibody testing, including data on their usual testing methods and practices. Study data were collected and managed using the REDCap online tool (https://projectredcap.org), hosted at the University of Bath [11]. A copy of the survey can be reviewed as supplementary material.

The survey was sent to all 530 IMACS members on 30 August 2019, and complete responses were received from 111 participants by the deadline of 30 September 2019. One hundred respondents were based at 65 institutions, across six continents. Eleven participants did not identify their institution and chose to complete the survey anonymously. Forty-two percent of respondents were based in USA/Canada, 21% in Europe, and 14% in Asia. Enzyme immunoassay/ELISA was reported to be the most widely used MSA detection technique worldwide and was used by 46% of respondents’ local laboratories. This was followed by the line blot, used by 37% of respondents’ laboratories. In Europe, the market was dominated by the Euroimmun line blot; 74% of European respondents’ local laboratories used a line blot and 48% the Euroimmun line blot. In USA/Canada, there was a more even spread of techniques used, with enzyme immunoassay/ELISA (36%), line blot (19%), and immunoprecipitation/immunoblot (23%) all popular testing methodologies. In Asia, 50% of respondents’ local laboratories utilized the line blot and 56% enzyme immunoassay/ELISA. Despite published concerns regarding the sensitivity/specificity of the various immunoassays, only 41% of respondents received guidance from their laboratory on the interpretation of positive results and 81% of participants stated that their laboratory failed to highlight discordant results obtained using multiple techniques (e.g., absence of cytoplasmic speckled staining on indirect immunofluorescence in association with a positive anti-synthetase antibody). This is a potential concern, as, with the more widespread availability of MSA detection methods, these tests are likely to be requested by non-expert users, who may lack the specialist knowledge required to interpret discordant or inconsistent results.

We found that 64% of the participants stated that they were confident with the results provided by their MSA testing laboratory. However, the same number of respondents also admitted that their confidence varied depending on the MSA in question, and this was largely due to concerns about false-positive and false-negative results. Despite these reservations, the majority of participants reported that MSA testing influenced their diagnostic confidence (83%), the information they relayed to their patients on prognosis (86%), further investigations planned (81%), and even their recommended treatment (73%). The latter finding was particularly surprising, given that the evidence base for pharmacological therapies in myositis is extremely limited, with support for the differential treatment response based on MSA positivity relying predominantly on the sub-analysis of a single randomized controlled trial [12, 13]. Key survey findings are summarized in Fig. 1.

**Fig. 1:**
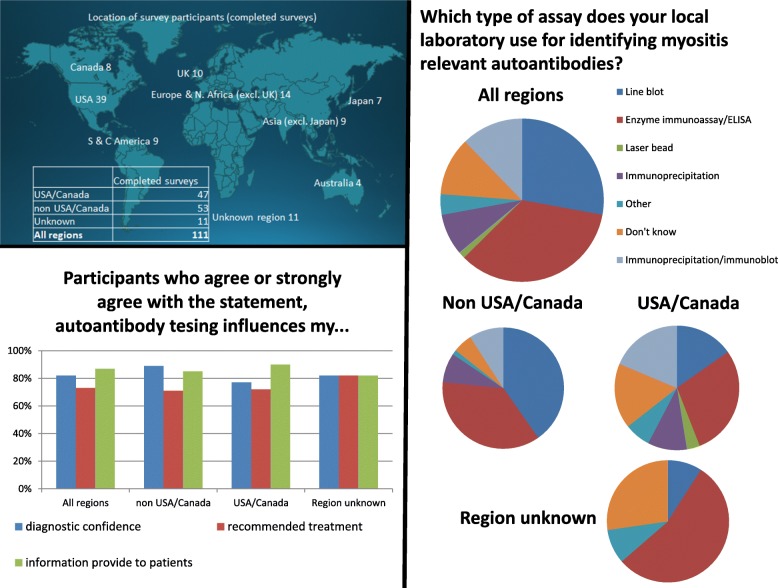
A visual summary of key survey findings

Our survey had several limitations, including a risk of bias and inaccuracy in data reporting (due to the possibility of participants completing the survey more than once and multiple participants from the same institution). Furthermore, the complex arrangements for autoantibody testing which exist at some institutions were not adequately captured by the survey. The survey predominantly captured the views of rheumatologists with an interest in myositis, who make up the majority of IMACS members (76% of respondents were rheumatologists, 8% neurologists, 5% dermatologists, 11% other medical specialties and anonymous respondents). These views may therefore not be shared by other medical professionals.

Our results clearly demonstrate that MSA testing by commercial immunoassays is being used globally to inform clinical decision-making. Although our survey respondents all have expertise in myositis, more than 90% felt that more education was needed on the interpretation of autoantibody results and this should be urgently addressed. We have identified the key MSA testing methods used globally (enzyme immunoassay/ELISA and line blot). While there may be some variation between manufacturers, we know that certain assay types perform poorly for particular myositis specificities, for example, in a significant proportion of patients, anti-TIF1ɣ autoantibodies target a conformational epitope and therefore will not be detected by blotting-based assays [9]. Our findings will allow a better targeting of resources and further research on test result accuracy and comparability, in addition to educational resources, which should focus the most commonly used assay types. Our data also provide some justification for comparing novel testing methods to other commercial assays which could be considered “standard clinical practice” although we would argue that immunoprecipitation as a gold standard method would be preferable where available.

Broader access to MSA testing is welcomed for its potential to improve patient outcomes. However, assessing the reliability of current testing methods is essential given the importance placed on MSA as clinical decision-making tools. An understanding of the limitations of the chosen testing method and guidance on when and in whom MSA tests should be performed is vital. This data justifies ongoing work to answer these important questions.

## Supplementary information

**Additional file 1.**

## Data Availability

The dataset created and analyzed during the current study is available from the corresponding author on reasonable request.
