# Supplementary material for: The promise, perceptions, and pitfalls of immunoassays for autoantibody testing in myositis
Source: Arthritis Res Ther. 2020 May 15;22:117. doi: 10.1186/s13075-020-02210-2 (PMC7227250; doi:10.1186/s13075-020-02210-2)
Supplement: Supplementary file 1 — Additional file 1. [file 13075_2020_2210_MOESM1_ESM.pdf]

Myositis Autoantibody Testing Survey

This survey aims to establish the availability and utilisation of different methods for identifying myositis-specific autoantibodies (MSA) within different units across the world.

International Myositis Assessment & Clinical Studies (IMACS) members from the MSA Special Interest Group and other IMACS members are being invited, via electronic invitation, to participate in this survey.

Results from the survey will help consolidate knowledge regards the current practice of myositis autoantibody testing, the types of assays that are being used, and the areas of uncertainty or gaps in knowledge that need addressing by future work.

The survey takes approximately 10 minutes to complete and this can be done at a time and place of your choosing.

Data management

This survey uses REDCap electronic data capture software. Data will be stored on a secure server at the University of Bath. Participants may choose to complete the survey anonymously or supply contact details to be acknowledged as a member of IMACS in any resulting publication. Aside from being acknowledged as a member of IMACS in any resulting publication (for consenting participants), personal information and responses from individual participants from the survey will remain confidential. Summary statistics for each question will be analysed and a summary report prepared. The aggregate results of the study may be printed/published or shared with other people in the scientific community.

Further information

Please contact the chief investigator, Professor Neil McHugh (n.j.mchugh@bath.ac.uk) if you require further clarification before deciding whether to participate. If you wish to independently discuss the purpose and/or conduct of this survey, please contact Professor James Betts, Chair of the Research Ethics Approval Committee for Health (REACH) via email (J.Betts@bath.ac.uk)

PART A. About you and your institution

Consent<br><br>You are not obliged to complete this survey and can withdraw at any stage, without explanation. Individual participant responses will remain confidential.<br><br>Please note that even if you opt to participate anonymously, it is possible (due to free text and/or low numbers) that your employer could identify you from your responses to the survey.<br><br>If you opt to enter your contact details, you have until 2 weeks after the final survey reminder to request that your survey data be deleted.<br><br>If you opt to participate anonymously, your contact details will not be requested and you will not be listed as a collaborator on any future publications. Affiliated institutions will only be included in outputs for participants consenting to be acknowledged.	◯ I consent to participate in this survey but would like to participate anonymously<br>◯ I consent to my participation in the study being acknowledged in any resulting publication<br>(By continuing with this survey I confirm that I have read and understood the consent information)
Title	◯ Dr.<br>◯ Prof.<br>◯ Mr.<br>◯ Mrs.<br>◯ Miss<br>◯ Ms.<br>◯ Other
Other title	__________________________________
First name	__________________________________
Surname	__________________________________
Email address	__________________________________
Institution	__________________________________
City	__________________________________

Country

◯ Afghanistan
◯ Aland Islands
◯ Albania
◯ Algeria
◯ American Samoa
◯ Andorra
◯ Angola
◯ Anguilla
◯ Antarctica
◯ Antigua and Barbuda
◯ Argentina
◯ Armenia
◯ Aruba
◯ Australia
◯ Austria
◯ Azerbaijan
◯ Bahamas
◯ Bahrain
◯ Bangladesh
◯ Barbados
◯ Belarus
◯ Belgium
◯ Belize
◯ Benin
◯ Bermuda
◯ Bhutan
◯ Bolivia
◯ Bonaire, Sint Eustatius and Saba
◯ Bosnia and Herzegovina
◯ Botswana
◯ Bouvet Island
◯ Brazil
◯ British Virgin Islands
◯ British Indian Ocean Territory
◯ Brunei Darussalam
◯ Bulgaria
◯ Burkina Faso
◯ Burundi
◯ Cambodia
◯ Cameroon
◯ Canada
◯ Cape Verde
◯ Cayman Islands
◯ Central African Republic
◯ Chad
◯ Chile
◯ China
◯ Hong Kong, Special Administrative Region of China
◯ Macao, Special Administrative Region of China
◯ Christmas Island
◯ Cocos (Keeling) Islands
◯ Colombia
◯ Comoros
◯ Congo (Brazzaville)
◯ Congo, Democratic Republic of the
◯ Cook Islands
◯ Costa Rica
◯ Côte d'Ivoire
◯ Croatia
◯ Cuba
◯ Curaçao
◯ Cyprus
◯ Czech Republic
◯ Denmark
◯ Djibouti
◯ Dominica
◯ Dominican Republic
◯ Ecuador
◯ Egypt

◯ El Salvador
◯ Equatorial Guinea
◯ Eritrea
◯ Estonia
◯ Ethiopia
◯ Falkland Islands (Malvinas)
◯ Faroe Islands
◯ Fiji
◯ Finland
◯ France
◯ French Guiana
◯ French Polynesia
◯ French Southern Territories
◯ Gabon
◯ Gambia
◯ Georgia
◯ Germany
◯ Ghana
◯ Gibraltar
◯ Greece
◯ Greenland
◯ Grenada
◯ Guadeloupe
◯ Guam
◯ Guatemala
◯ Guernsey
◯ Guinea
◯ Guinea-Bissau
◯ Guyana
◯ Haiti
◯ Heard Island and Mcdonald Islands
◯ Holy See (Vatican City State)
◯ Honduras
◯ Hungary
◯ Iceland
◯ India
◯ Indonesia
◯ Iran, Islamic Republic of
◯ Iraq
◯ Ireland
◯ Isle of Man
◯ Israel
◯ Italy
◯ Jamaica
◯ Japan
◯ Jersey
◯ Jordan
◯ Kazakhstan
◯ Kenya
◯ Kiribati
◯ Korea, Democratic People's Republic of
◯ Korea, Republic of
◯ Kuwait
◯ Kyrgyzstan
◯ Lao PDR
◯ Latvia
◯ Lebanon
◯ Lesotho
◯ Liberia
◯ Libya
◯ Liechtenstein
◯ Lithuania
◯ Luxembourg
◯ Macedonia, Republic of
◯ Madagascar
◯ Malawi
◯ Malaysia
◯ Maldives
◯ Mali
◯ Malta
◯ Marshall Islands

◯ Martinique
◯ Mauritania
◯ Mauritius
◯ Mayotte
◯ Mexico
◯ Micronesia, Federated States of
◯ Moldova
◯ Monaco
◯ Mongolia
◯ Montenegro
◯ Montserrat
◯ Morocco
◯ Mozambique
◯ Myanmar
◯ Namibia
◯ Nauru
◯ Nepal
◯ Netherlands
◯ Netherlands Antilles
◯ New Caledonia
◯ New Zealand
◯ Nicaragua
◯ Niger
◯ Nigeria
◯ Niue
◯ Norfolk Island
◯ Northern Mariana Islands
◯ Norway
◯ Oman
◯ Pakistan
◯ Palau
◯ Palestinian Territory, Occupied
◯ Panama
◯ Papua New Guinea
◯ Paraguay
◯ Peru
◯ Philippines
◯ Pitcairn
◯ Poland
◯ Portugal
◯ Puerto Rico
◯ Qatar
◯ Réunion
◯ Romania
◯ Russian Federation
◯ Rwanda
◯ Saint-Barthélemy
◯ Saint Helena
◯ Saint Kitts and Nevis
◯ Saint Lucia
◯ Saint-Martin (French part)
◯ Saint Pierre and Miquelon
◯ Saint Vincent and Grenadines
◯ Samoa
◯ San Marino
◯ Sao Tome and Principe
◯ Saudi Arabia
◯ Senegal
◯ Serbia
◯ Seychelles
◯ Sierra Leone
◯ Singapore
◯ Sint Maarten (Dutch part)
◯ Slovakia
◯ Slovenia
◯ Solomon Islands
◯ Somalia
◯ South Africa
◯ South Georgia and the South Sandwich Islands
◯ South Sudan
◯ Spain

◯ Sri Lanka
◯ Sudan
◯ Suriname *
◯ Svalbard and Jan Mayen Islands
◯ Swaziland
◯ Sweden
◯ Switzerland
◯ Syrian Arab Republic (Syria)
◯ Taiwan
◯ Tajikistan
◯ Tanzania *, United Republic of
◯ Thailand
◯ Timor-Leste
◯ Togo
◯ Tokelau
◯ Tonga
◯ Trinidad and Tobago
◯ Tunisia
◯ Turkey
◯ Turkmenistan
◯ Turks and Caicos Islands
◯ Tuvalu
◯ Uganda
◯ Ukraine
◯ United Arab Emirates
◯ United Kingdom
◯ United States of America
◯ United States Minor Outlying Islands
◯ Uruguay
◯ Uzbekistan
◯ Vanuatu
◯ Venezuela (Bolivarian Republic of)
◯ Viet Nam
◯ Virgin Islands, US
◯ Wallis and Futuna Islands
◯ Western Sahara
◯ Yemen
◯ Zambia
◯ Zimbabwe

State

- ◯ Alabama
- ◯ Alaska
- ◯ Arizona
- ◯ Arkansas
- ◯ California
- ◯ Colorado
- ◯ Connecticut
- ◯ Delaware
- ◯ District of Columbia
- ◯ Florida
- ◯ Georgia
- ◯ Hawaii
- ◯ Idaho
- ◯ Illinois
- ◯ Indiana
- ◯ Iowa
- ◯ Kansas
- ◯ Kentucky
- ◯ Louisiana
- ◯ Maine
- ◯ Maryland
- ◯ Massachusetts
- ◯ Michigan
- ◯ Minnesota
- ◯ Mississippi
- ◯ Missouri
- ◯ Montana
- ◯ Nebraska
- ◯ Nevada
- ◯ New Hampshire
- ◯ New Jersey
- ◯ New Mexico
- ◯ New York
- ◯ North Carolina
- ◯ North Dakota
- ◯ Ohio
- ◯ Oklahoma
- ◯ Oregon
- ◯ Pennsylvania
- ◯ Rhode Island
- ◯ South Carolina
- ◯ South Dakota
- ◯ Tennessee
- ◯ Texas
- ◯ Utah
- ◯ Vermont
- ◯ Virginia
- ◯ Washington
- ◯ West Virginia
- ◯ Wisconsin
- ◯ Wyoming

How many patients are in your local myositis cohort?	__________________________________
What is your professional role?	◯ Clinician<br>◯ Non-clinical scientist<br>◯ Clinical trainee<br>◯ Post-doctoral research fellow<br>◯ Other (please describe)
Other role details	__________________________________
Are you a full member of the International Myositis Assessment & Clinical Studies (IMACS) Group?	◯ Yes<br>◯ No
Are you a member of the Myositis-Specific Autoantibodies (MSA) Special Interest Group (SIG)?	◯ Yes<br>◯ No

PART B. Myositis autoantibody testing

Q1. Which type of assay does your local laboratory use for identifying myositis relevant autoantibodies?

☐ Line blot
☐ Enzyme immunoassay/ELISA
☐ Laser bead
☐ Immunoprecipitation
☐ Immunoprecipitation/immunoblot
☐ Other (please state below)
☐ Don't know
(Select all that apply)

Line blot manufacturer (if known) __________________________________

EIA manufacturer (if known) __________________________________

Laser bead manufacturer (if known) __________________________________

Immunoprecipitation provider (if known) __________________________________

Immunoprecipitation/immunoblot provider (if known) __________________________________

Other assay type __________________________________

Q2. Do you send serum to another collaborating institution for antibody testing?

- ◯ Yes
- ◯ No

How many other laboratories do you use?

- ◯ 1
- ◯ 2
- ◯ 3 or more

Other laboratory 1 details

____
(Name or location of main collaborating institution)

Which type of assay does your collaborating institution laboratory use for identifying myositis relevant autoantibodies?

- ☐ Line blot
- ☐ Enzyme immunoassay/ELISA
- ☐ Laser bead
- ☐ Immunoprecipitation
- ☐ Immunoprecipitation/immunoblot
- ☐ Other (please state below)
- ☐ Don't know

(Select all that apply)

Line blot manufacturer (if known)

____

EIA manufacturer (if known)

____

Laser bead manufacturer (if known)

____

Immunoprecipitation provider (if known)

____

Immunoprecipitation/immunoblot provider (if known)

____

Other assay type (and manufacturer if known)

____

Other laboratory 2 details

____

Which type of assay does your collaborating institution laboratory use for identifying myositis relevant autoantibodies?

- ☐ Line blot
- ☐ Enzyme immunoassay/ELISA
- ☐ Laser bead
- ☐ Immunoprecipitation
- ☐ Immunoprecipitation/immunoblot
- ☐ Other (please state below)
- ☐ Don't know

(Select all that apply)

Line blot manufacturer (if known)

____

EIA manufacturer (if known)

____

Laser bead manufacturer (if known) __________________________________

Immunoprecipitation provider (if known) __________________________________

Immunoprecipitation/immunoblot provider (if known) __________________________________

Other assay type (and manufacturer if known) __________________________________

Other laboratory 3 details __________________________________

Which type of assay does your collaborating institution laboratory use for identifying myositis relevant autoantibodies?

- ☐ Line blot
- ☐ Enzyme immunoassay/ELISA
- ☐ Laser bead
- ☐ Immunoprecipitation
- ☐ Immunoprecipitation/immunoblot
- ☐ Other (please state below)
- ☐ Don't know

(Select all that apply)

Line blot manufacturer (if known) __________________________________

EIA manufacturer (if known) __________________________________

Laser bead manufacturer (if known) __________________________________

Immunoprecipitation provider (if known) __________________________________

Immunoprecipitation/immunoblot provider (if known) __________________________________

Other assay type (and manufacturer if known) __________________________________

Q3. Is indirect immunofluorescence performed on all sera as part of myositis autoantibody identification?

- ◯ Yes
- ◯ No
- ◯ Don't know

Q4. In which patients would you typically test for myositis relevant autoantibodies?

- ☐ A. Raised serum CpK in isolation
- ☐ B. Raised serum CpK in presence of other features of myositis spectrum disorders
- ☐ C. Symptoms and signs of inflammatory arthritis/arthralgia in isolation
- ☐ D. Symptoms and signs of inflammatory arthritis/arthralgia in presence of other features of myositis spectrum disorders
- ☐ E. Fever in isolation
- ☐ F. Fever in presence of other features of myositis spectrum disorders
- ☐ G. Possible DM rash in isolation
- ☐ H. Possible DM rash in presence of other features of myositis spectrum disorders
- ☐ I. Raynaud's in isolation
- ☐ J. Raynaud's in the presence of other features of myositis spectrum disorders
- ☐ K. Cytoplasmic speckle identified on indirect immunofluorescence alone
- ☐ L. Cytoplasmic speckle identified on indirect immunofluorescence in presence of other features of myositis spectrum disorders
- ☐ M. Interstitial lung disease in isolation
- ☐ N. Interstitial lung disease in presence of other features of myositis spectrum disorders
- ☐ O. Clinical symptoms and signs suggestive of SLE
- ☐ P. Clinical symptoms and signs suggestive of systemic sclerosis
- ☐ Q. Other (please describe)

(Select all that apply)

Other criteria

____

Q5.	Does your local laboratory report include?	☐ A. Positive vs negative result<br>☐ B. Semi-quantification of a positive result e.g. strong, weak etc.<br>☐ C. Guidance on interpretation of positive results<br>(Select all that apply)
	If C selected, is this adequate for your needs?	◯ Yes<br>◯ No

Q6. Does your local laboratory highlight discordant results as part of their report? E.g. ANA pattern does not match antibody identified.

◯ Yes
◯ No
◯ Don't know

Confidence in reported results of local laboratory

Q7a.	In general, I am confident in the result obtained from my local laboratory	◯ Strongly disagree<br>◯ Disagree<br>◯ Neither agree nor disagree<br>◯ Agree<br>◯ Strongly agree
Q7b.	Does your confidence vary depending on the autoantibody?	◯ Yes<br>◯ No
	If yes, are there certain assays that you have concerns related to either false positives or false negatives?	◯ Yes<br>◯ No
	If yes, please describe	____

Q8. Impact of autoantibody testing

	Strongly disagree	Disagree	Neither agree nor disagree	Agree	Strongly agree
Q8a. Autoantibody testing influences my diagnostic confidence	○	○	○	○	○
Q8b. Autoantibody testing influences the information I provide to a patient on prognosis	○	○	○	○	○
Q8c. Autoantibody testing influences any further investigations I arrange	○	○	○	○	○
Q8d. Autoantibody testing influences my recommended treatment	○	○	○	○	○

Q8e. Any additional comments? ______________________________

Q9.	Do you think there is a need for more education on how to interpret autoantibody results?	◯ Yes<br>◯ No
	If yes, please select potentially useful strategies for providing education	☐ A. IMACS website<br>☐ B. EULAR course<br>☐ C. Other (please describe)<br>(Select all that apply)
	Other strategy	______________________
